# Reproducibility of double agar gel immunodiffusion test using stored serum and plasma from paracoccidioidomycosis patients

**DOI:** 10.1590/1678-9199-JVATITD-2022-0045

**Published:** 2023-01-16

**Authors:** Karina Andressa Tomazini, Beatriz Aparecida Soares Pereira, Tatiane Fernanda Sylvestre, Ricardo de Souza Cavalcante, Lídia Raquel de Carvalho, Rinaldo Poncio Mendes

**Affiliations:** 1 Department of Infectology, Dermatology, Diagnostic Imaging and Radiotherapy, Botucatu Medical School (FMB), São Paulo State University (UNESP), Botucatu, SP, Brazil.; 2 Anhanguera School of Bauru, Bauru, SP, Brazil.; 3 Department of Biodiversity and Biostatistics, Institute of Biosciences, São Paulo State University (UNESP), Botucatu, SP, Brazil.

**Keywords:** Paracoccidioidomycosis, Serum, Plasma, Storage, Serological diagnosis, Blood matrices

## Abstract

**Background:**

Serological evaluation performed by double agar gel immunodiffusion test (DID) is used for diagnosis, evaluation of severity, management of paracoccidioidomycosis patients, and development of new clinical studies. For these reasons, the Botucatu Medical School of UNESP maintains a serum bank at the Experimental Research Unit with patient clinical data. This study aimed to evaluate the influence of the freeze-thaw cycle and different blood matrices on the titration of circulating antibodies.

**Methods:**

The study included 207 patients with confirmed (etiology-demonstrated) or probable (serology-demonstrated) paracoccidioidomycosis, and DID was performed with culture filtrate from *Paracoccidioides brasiliensis* B339 as antigen. First experiment: the antibody levels were determined in serum samples from 160 patients with the chronic form and 20 with the acute/subacute form, stored at -80^o^C for more than six months. Second experiment: titers of 81 samples of serum and plasma with ethylenediaminetetraacetic acid (EDTA) or heparin, from 27 patients, were compared according to matrix and effect of storage at -20^o^C for up to six months. Differences of titers higher than one dilution were considered discordant.

**Results:**

First experiment: test and retest presented concordant results in serum stored for up to three years, and discordant titers in low incidence in storage for four to six years but high incidence when stored for more than six years, including conversion from reagent test to non-reagent retest. Second experiment: serum, plasma-EDTA and plasma-heparin samples showed concordant titers, presenting direct correlation, with no interference of storage for up to six months.

**Conclusions:**

Storage at -80^o^C for up to six years has no or little influence on the serum titers determined by DID, permitting its safe use in studies depending on this parameter. The concordant titrations in different blood matrices demonstrated that the plasma can be used for immunodiffusion test in paracoccidioidomycosis, with stability for at least six months after storage at -20^o^C.

## Background

Paracoccidioidomycosis (PCM) is a systemic mycosis caused by thermodimorphic fungi of the genus *Paracoccidioides* [1]. Confined to Latin America, it is endemic in an area that extends from Mexico to Argentina [2], with a higher incidence in Brazil, where it is diagnosed with great frequency in São Paulo state [3]. PCM is observed in patients such as rural workers who have been or are in direct and prolonged contact with the soil [3,4,5]. It predominates in males, with a masculinity ratio of 1.7:1.0 in the acute/subacute form and 22.0:1.0 in the chronic form, being more prevalent in the age group between 30 and 59 years old [6]. Its diagnosis is made by identifying the typical forms of the yeast phase of *P. brasiliensis* in clinical materials such as sputum, bronchoalveolar lavage, lymph node, skin and mucosal lesion scraping and tissue fragments, exceptionally in cerebrospinal fluid (CSF) and rarely in blood [7]. 

The double agar gel immunodiffusion test (DID) is used in PCM for diagnosis due to its adequate sensitivity (90%) and high specificity(100%) [6]; evaluation of severity because of the direct correlation between its serological titer and the degree of the patient severity [8]; and control of cure due to the correlation between the decreasing serological titer, improvement of the clinical conditions [9], decreasing IL-10 production and increasing IFN-ϒ and IL-2 production [10] after appropriate treatment [8]. In areas with predominance of fungi from the *Paracoccidioides brasiliensis* complex, the antigen used for detection of the specific antibodies is the culture filtrate of the strain B339, which is rich in gp43 [6]. In addition, the evaluation of the shelf life of biological samples serologically reactive for *Paracoccidioides* spp. is also important for studies in standardization, validation, and optimization of serological assay, as well as for the evaluation of new antigens. 

A biobank with serum samples was organized aiming at the reevaluation of the antibody levels when a result showed no compatibility with the clinical evaluation and/or with other laboratory findings. As the follow-up was performed at progressive intervals - initially monthly, then every three and finally every six months - this is the longest time of the freeze-thaw cycle between two consecutive appointments. In this case, the question was a possible influence of this cycle on the reproducibility of the titration. In addition, this storage of serum could be used in several studies, since careful clinical and other laboratory evaluations were simultaneously registered. The serum bank is particularly useful in studies on PCM because the number of new cases from Botucatu region (São Paulo state, Brazil) is about 15 per year [6] and the follow-up is carried out until the apparent cure, which is reached in about two years for patients with the acute/subacute form (AF) and four years in those with the chronic form (CF). Thus, the knowledge of the reproducibility of the titration after a freeze-thaw cycle is essential, however, it was performed in two studies in patients with candidiasis and in one research carried out in patients with allergic bronchopulmonary aspergillosis [11,12,13].

Moreover, there are no studies evaluating other blood matrices to be used in the determination of circulating antibodies by DID in PCM-patients, nor its reproducibility after the freeze-thaw cycle. The serum is obtained putting the blood in tubes with activators of the coagulation - as silica (dioxide of silicon), and the plasma placing in tubes with etylenodiaminotetracetic acid (EDTA), heparin or sodium citrate [14,15]. As a consequence of such preparations, the serum presents a higher fragmentation of the proteins, forming aminoacids, which leads to a lower molecular weight and a higher concentration of metabolites, while the plasma maintains the coagulation factors, preserved proteins, hormones and electrolytes [16, 17]. 

The present study was carried out to evaluate the reproducibility of the antibody levels in serum from PCM-patients stored at -80^o^C from six months to 30 years, the comparison of the titers determined in different blood matrices, and finally the reproducibility of the titration in serum and plasma samples stored at -20^o^C for up to six months. 

## Methods

### Patients

This study was carried out in 207 patients with active PCM seen at the University Hospital of the São Paulo State University (UNESP), in the Botucatu Medical School, a tertiary hospital with 490 beds distributed among all the specialities, which receives patients from 68 municipalities of the Regional Health Board VI (São Paulo state, Brazil). These patients were seen at the Clinical Mycology Outpatient service.

Confirmed cases, characterized by identification of the etiologic agent in clinical materials, and probable cases, defined only by demonstration of the specific serum antibodies determined by the double agar gel immunodiffusion test (DID) were included in the study. Patients with comorbidity of infectious, inflammatory non-infectious or neoplastic origin, except smoking habit and alcohol intake were excluded. 

The clinical form of these patients was classified according to Mendes et al. [18] specifications, updated by Mendes et al. [8]. Only one blood sample was evaluated from each patient. 

### Study design

**Figure 1.**
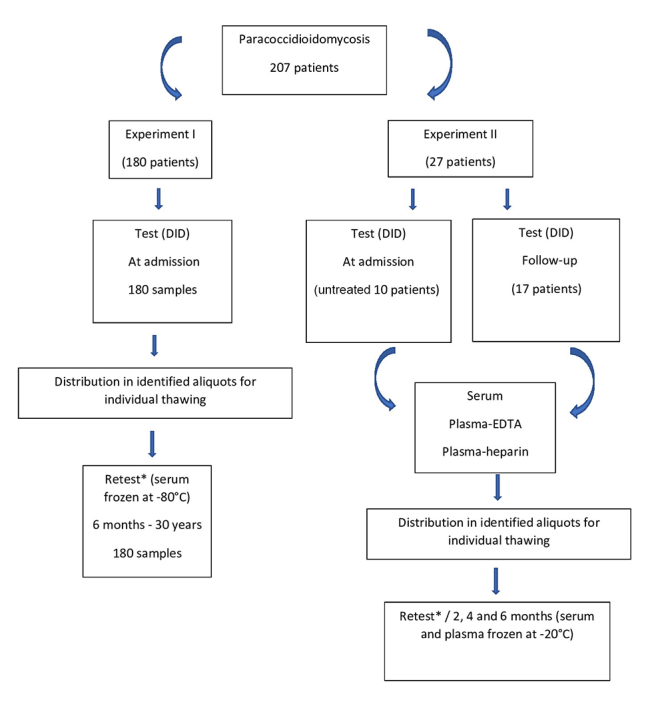
Study design to evaluate the anti-*Paracoccidioides brasiliensis* circulating antibodies determined by the double agar gel immunodiffusion test in serum and plasma obtained with ethylenodiaminetetracetic acid (EDTA) or heparin. Influence of freezing on different blood matrices, stored in aliquots to be thawed only once, on the titration of the antibodies. *The retest was performed in the same sample used in the test.

This study comprised two experiments - the first, to analyse the effect of storage at -80^o^C on the titers of the specific serum antibodies, and the second to evaluate the influence of the blood matrix and of the storage at -20^o^C on these determinations (Figure 1).

### Sample size

The sample size calculation for the experiments I and II was performed considering the use of paired ***t*** test, before and after storage, the type I error of 0.05 and a difference of at least two dilutions between titrations, according to the specifications by Zar [19]. As a result, the sample size should be of at least nine. 

### Experiment I

A total of 180 patients with active PCM, 20 of whom with the acute/subacute form (AF) and 160 with the chronic form (CF), were evaluated.

The blood samples from these patients were drawn at admission and the specific serum levels were determined by the double agar gel immunodiffusion test (DID). Then, the serum samples were frozen and maintained at -80^o^C for variable periods between six months and 30 years, in the biobank of the Experimental Research Unit (Unipex), Botucatu Medical School of UNESP. The storage was performed in small serum aliquots that were discarded after use. The choice of patients was based on the volume of serum available in the biobank, independently of the storage time.

Experiment I consisted in the comparison of the specific antibody serum levels determined at admission, identified as **test**, with those obtained after a freeze-thaw cycle, identified as **retest**.

### Experiment II

A prospective study of 27 patients with active PCM - 22.2% with AF and 71.8% with CF was carried out; 10 patients were evaluated at admission and 17 during the follow-up of the treatment. This study consisted of the comparison of the serum and plasma levels of the specific antibodies determined by DID test and the effect of the storage at -20^o^C for two (stage 1), four (stage 2), and six months (stage 3). The circulating specific antibody levels determined at the inclusion in the study were identified as stage 0.

### Clinical samples

Serum and plasma obtained with ethylenediaminetetraacetic acid (EDTA) or with heparin were used in the present study. The serum samples were collected in tubes with coagulum activator, silica and separator gel (5-mL *vacuplast*), and the plasma samples in tubes with EDTA (4-mL *vacuplast*) or with heparin (4-mL *vacuplast*). All the samples were divided into aliquots, and then stored at -20^o^C. After usage, they were discarded, in order to perform only one titration after thawing.

### Determination of the specific circulating antibodies

The serum and plasma levels of the anti-*P. brasiliensis* antibodies were determined by the double agar gel immunodiffusion test (DID), according to Restrepo [20], in the Laboratory of Tropical Diseases, Unipex (UNESP, Botucatu).

The culture filtrate of the leveduriform phase of *P. brasiliensis* strain B339 was the antigen used. Briefly, *P. brasiliensis* strain B339 was cultivated in Fava Netto medium at 35^o^C for five days; then it was inoculated in Negroni medium modified by Siqueira and incubated in a shaking at 35^o^C for 15 days. Next, thimerosal was added to the final concentration of 0.2g/mL, maintaining the incubation in the same conditions for another four days. The antigen was filtrated, concentrated 10 times with polyethylene glycol (Sigma P-2263), and dialyzed against phosphate-buffered saline (PBS) in a dialysis membrane (Sigma D-9377). The concentrate extract was evaluated according to its protein content, analyzed by SDS-PAGE, aliquoted, and frozen at -70^o^C.

 In the experiment I the antigen used in the test was prepared in the Department of Clinical Analyses, School of Pharmaceutical Sciences of Araraquara, Prof. Maria José Soares Mendes Giannini service, and the antigen used in the retest was isolated from the same strain and produced according to the same specifications, but in the Laboratory of Tropical Diseases, Unipex (UNESP, Botucatu). Therefore, in experiment I the antigen batch and the technician were not the same while in experiment II they were the same. In each titration, a reagent and a non-reagent serum were used as controls. 

The serum and plasma samples were initially tested undiluted and, then, diluted from ½, with a ratio of 2.0. Discordance of titers between test and retest, as well as between blood matrices were considered when higher than one dilution. Thus, the titers were classified into concordant, when exactly the same or different in only one dilution and discordant, when different in at least two dilutions. The titers were also classified according to its intensity - mild from 1 to 8, moderate when equal to 16 or 32, and intense when ≥ 64. 

### Statistical analysis

Data were analyzed in order to evaluate the effect of the storage and the blood matrices on the titration of circulating anti-*P. brasiliensis* antibodies. To perform the statistical analysis, the titers, characterized as the denominator of the dilution, were converted in scores (Table 1). The reproducibility of the titers was evaluated in both a quantitative manner - comparing medians, and in a qualitative one, i.e., reagent *versus* non-reagent.

**Table 1.** Determination of the circulating anti-*Paracoccidioides brasiliensis* antibodies by the double agar gel immunodiffusion test. Conversion in scores of non-reagents and reagents as to dilution.

Titer*	Score	Titer	Score	Titer	Score
Non-reagent	0	8	4	128	8
1 (reagent undiluted)	1	16	5	256	9
2	2	32	6	512	10
4	3	64	7	1.024	11

*Titer expressed as the denominator of the dilution.

The results were presented as median, 1^st^ and 3^rd^ quartiles. The comparison of paired continuous variables was performed by Wilcoxon match-pairs signed-rank test or by Friedman test, and of the independent ones by Kruskal-Wallis test. The categorical variables were compared by chi-square test or Fisher’s exact test or by binomial test, by Goodman test or by Marascuilo procedure. The correlation between variables was evaluated according to Spearman. Significance was set up at p ≤ 0.05. The statistical analyses were performed using the Statistical Analyses System (SAS), version 9.2.

### Ethics research committee

This study was approved by the Ethics Research Committee of the São Paulo State University (UNESP), Botucatu Medical School, protocol number 51849721.7.0000.5411.

## Results

The results of experiments I and II will be presented separately.

### Experiment I

The intensity of the antibody serum levels determined at admission (test) did not vary in patients with the acute form (AF), and it was practically constant in those with the chronic form (CF). Similar findings were observed in determinations after storage at -80^o^C (Additional file 1).

The comparison between titers of the test and retest differed in only one dilution, with decrease after storage in patients with the AF, and increase in those with the CF. The evaluation of all the samples showed no difference between test and retest (Table 2).

**Table 2.** Titers of specific serum antibodies determined by double agar gel immunodiffusion test, in samples from 20 patients with the acute/subacute form and 160 with the chronic form of paracoccidioidomycosis. Comparison of the titers determined at the moment of blood draw (test) and after at least six months of storage at -80^o^C (retest), presented as median, 1^st^, and 3^rd^ interquartile, Wilcoxon rank test.

Clinical form	Patients (number)	Test (titer)	Retest (titer)	***p* value**
Acute/subacute	20	128 (32; 512)	64 (4; 512)	0.01
Chronic	160	16 (4; 64)	32 (2; 128)	< 0.001
Total	180	16 (4; 64)	8 (2; 64)	0.14

The distribution of the serum as to the titration of the specific antibodies showed higher prevalence of intense levels in the AF; in the CF the prevalence of the mild titers were higher than the intense ones, with the moderate occupying an intermediate position. This distribution was altered in the titration after storage at -80^o^C, with prevalence presenting only a tendency to be different, in the AF as well as in the CF (Table 3). 

**Table 3.** Distribution of 20 patients with the acute/subacute form and 160 with the chronic form of paracoccidioidomycosis as to the intensity of the specific antibody serum levels, determined by the double agar gel immunodiffusion test, clinical form and moment of the serological evaluation - at admission (test) and after at least six months of storage at -80^o^C (retest).

Evaluation	Clinical form	Mild 79 (100)	Moderate 45 (100)	Intense 56 (100)	**Value of *p***	Interpretation
Test	Acute/subacute n = 20	03 (15.0)	03 (15.0)	14 (70.0)	< 0 .001	I > (M = Mo)
	Chronic n = 160	76 (47.5)	42 (26.25)	42 (26.25)		M > I; Mo Intermediary

Retest	Acute/subacute (n = 20)	8 (40.0)	1 (5.0)	11 (55.0)	0.05 < p < 0.10	...
	Chronic	56 (35.0)	37 (23.1)	67 (41.9 )	(tendency)	...

M: mild - titers up to 8; Mo: moderate - titers equal to 16 and 32; I: intense - titers ≥ 64; number in parentheses ( ): percentage; ...: absence of data.

The comparison between clinical forms demonstrated that there is no difference regarding the higher titer - test or retest and prevalence of discordant values, based on the difference of at least two dilutions (Table 4). In addition, the analysis of discordant values based on the difference of at least three dilutions showed a tendency to be higher in the AF than in the CF (***p*** = 0.07). 

**Table 4.** Distribution of 20 patients with the acute/subacute form and 160 with the chronic form of paracoccidioidomycosis as to the intensity of the specific antibody serum levels, determined by the double agar gel immunodiffusion test, at the moment of blood draw (test - T) and after at least six months of storage at -80^o^C (retest - Rt). Comparisons performed by chi-square test or by Fisher’s exact test.

	AF (n = 20)			CF (n = 160)		
	T = Rt	T > Rt	Rt > T	T = Rt	T > Rt	Rt > T
Equal titers	2	-	-	30	-	-
Different titers						
One dilution		5	4		21	50
Two dilutions		-	4		15	19
Three dilutions		1	-		4	-
Four dilutions		1	-		8	1
Five dilutions		1	-		5	-
Six dilutions		1	-		-	-
Seven dilutions		-	-		3	-
Eight dilutions		1	-		4	-
Total	2 (10.0)	10 (50.0)	8 (40.0)	30 (18.75)	60 (37.50)	70 (43.75)

AF: acute/subacute form; CF: chronic form.

Comparison between clinical forms

a) as a function of the highest titer (T or Rt): chi-square test

p > 0.05 AF = CF 

b) depending on the prevalence of disagreements (≥ 2 dilutions)

 AF = 45%; CF = 37%; ***p*** = 0.48; AF = CF

c) depending on the prevalence of disagreements (≥ 3dilutions)

 AF = 25.0%; CF = 15.6%; ***p*** = 0.07; AF = CF 

**Figure 2.**
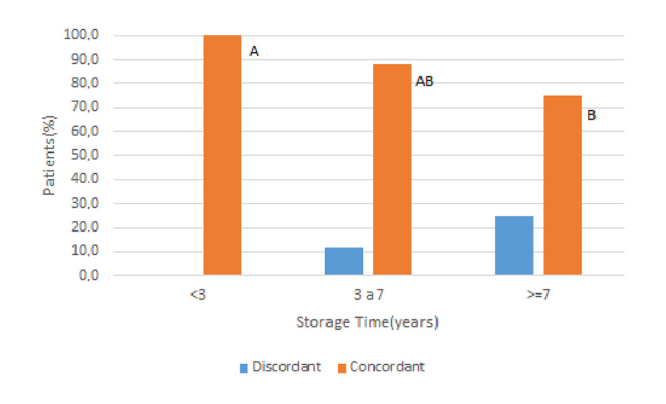
Evaluation of 180 paracoccidioidomycosis patients according to the percentual of concordant titers of anti-*P. brasiliensis* serum levels determined by the double agar gel immunodiffusion test and time of storage at -80^o^C. Goodman test (***p*** = 0.003). Titers are considered concordant if the difference between test and retest is of only one dilution. Frequencies followed by the same letter do not differ among them (***p*** > 0.05), while those followed by different letters present statistically significant differences (***p*** ≤ 0 .05).

Moreover, the prevalence of concordant titers regarding the length of the storage showed a decrease in serum samples stored for more than six years (Figure 2). 

The analysis of 45 serum samples stored for three to six years showed that 14 (31.1%) presented the same titration, 18 (40%) differed in one dilution, 12 (26.7%) in two dilutions, and only 1 (2.2%) in three dilutions. Therefore, in this sample, concordant titers were observed in 32 (71.1%) and discordant in 13 (28.9%) sera. In addition, the distribution of the storage time, in years, running between test and retest, presented as median, first, and third quartiles was not different (***p*** = 0.85) among groups in which the titers were the same [4.4 (3.5; 5.8)], different in one dilution [4.0 (3.7; 5.5)], or in two dilutions [4.5 (3.4; 5.5)].

Finally, the conversion from reagent in the test to non-reagent in the retest of sera stored for more than six years at -80^o^C was observed in 20.0% of those from patients with the AF (MacNemar test, p = 0.03), and in 16.3% with the CF (MacNemar test, p < 0.001). These rates of conversion as to clinical form were not different (chi-square test; AF = CF; ***p*** = 0.14).

### Experiment II

There was no difference between the serum and plasma antibody anti-*P. brasiliensis* titers determined by the double agar gel immunodiffusion test, in the evaluations at admission (before the introduction of the therapy) as well as in patients under treatment. In addition, the storage of these blood matrices at -20^o^C for two to six months did not influence the titers determined previously (Table 5).

**Table 5.** Serum and plasma titers of anti-*Paracoccidioides brasiliensis* antibodies determined by the double agar gel immunodiffusion test in blood samples drawn from 27 paracoccidioidomycosis patients, in specific tubes. Comparison of the titers observed in different blood matrices, in 10 patients at admission, before treatment (BT), and in 17 patients under antifungal treatment (T), at the moment of blood draw and after two to six months of storage at -20^o^C

5A	BT (n = 10)	T (n = 17)	5B	BT (n = 10)	T (n = 17)
Silica-serum	4 (NR; 64)	2 (NR; 4)		4 (2; 128)	2 (1; 4)
EDTA-plasma	8 (ND; 128)	2 (ND; 4)		16 (2; 128)	2 (1; 4)
Heparin-plasma	8 (4; 128)	2 (ND; 2)		16 (2; 256)	2 (1; 4)
***p***	0.75	0.97		0.93	0.98

**5C**	**BT (n = 10)**	**T (n = 17)**	**5D**	**BT (n = 10)**	**T (n = 17)**
Silica-serum	16 (4; 128)	4 (1; 8)		16 (2; 128)	2 (1; 4)
EDTA-plasma	8 (0; 32)	2 (1; 8)		32 (2; 128)	2 (1; 4)
Heparin-plasma	16 (0; 64)	2 (1; 8)		32 (1; 128)	2 (1; 4)
***p***	0.88	0.77		0.95	0.98

5A: At the moment of blood draw, a fresh sample (stage S_0_); 5B: after two months of storage (stage S_1_); 5C: after four months of storage (stage S_2_); 5D: after six months of storage (stage S_3_). Data presented as median, 1^st^ and 3^rd^ quartiles, Kruskal-Wallis test. NR: non reagent; ND: non diluted.

The titers did not alter regarding the time of storage at -20^o^C, in samples from patients evaluated at admission as well as those from patients under antifungal treatment, in the different blood matrices used (Table 6). 

**Table 6.** Titers of anti-*Paracoccidioides brasiliensis* antibodies determined by the double agar gel immunodiffusion test in serum (S-S), plasma obtained with EDTA (P-E) and plasma obtained with heparin (P-H). Samples from 27 paracoccidioidmycosis patients, namely 10 untreated (BT) and 17 under antifungal therapy (T). Analysis of the variation of the titers as to the time of storage at -20^o^C. Data presented as median, 1^st^ and 3^rd^ quartiles, Friedman test.

	S-S	BT n = 10	T n = 17	P-E	BT n = 10	T n = 17	P-H	BT n = 10	T n = 17
E_0_		4 (0; 64)	2 (0; 4)		8 (1; 128)	2 (1; 4)		8 (4; 128)	2 (1; 2)
E_1_		4 (2; 128)	2 (1; 4)		16 (2; 128)	2 (1; 4)		16 (2; 256)	2 (1; 4)
E_2_		16 (4; 128)	4 (1; 8)		8 (0; 32)	2 (1; 8)		16 (0; 64)	2 (1; 8)
E_3_		16 (2; 128)	2 (1; 4)		32 (2; 128)	2 (1; 4)		32 (1; 128)	2 (1; 4)
*p*		0.94	0.51		0.59	0.92		0.48	0.32

Pre-storage: E0; after-storage: E1 - 2 months; E2 - 4 months; E3 - 6 months.

**Figure 3.**
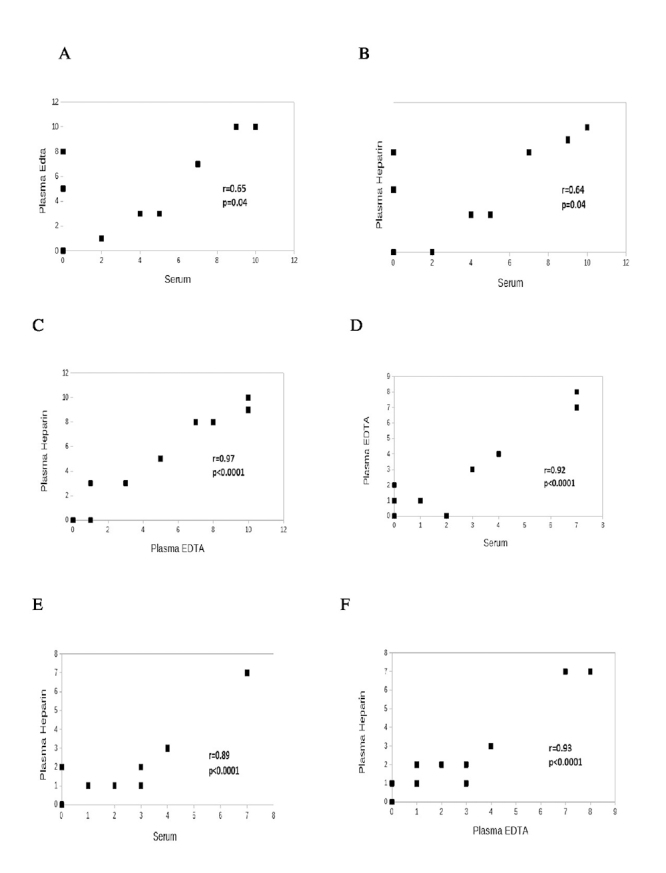
Correlations between serum and plasma levels of anti-*Paracoccidioides brasiliensis* antibodies determined by double agar gel immunodiffusion test in 27 patients with paracoccidioidomycosis. Correlations observed in **(A, B, C)** 10 patients before treatment and in **(D, E, F)** 17 patients under treatment follow-up. Plasma-EDTA: plasma obtained with ethylenediaminetetraacetic acid; plasma-heparin: plasma obtained with heparin. Spearman correlation test.

The titers determined in the three matrices showed direct correlation in all the stages - at the moment the blood was drawn (S_0_) and after two (S_1_), four (S_2_), and six months (S_3_) of storage at -20^o^C (Figure 3).

## Discussion

Serum storage from PCM patients under treatment has as its basic objective the repetition of the determination of the antibody titers when its results are not compatible with the clinical evaluation and/or other complementary evaluations - biochemical, hematological, or radiological. As these patients’ reevaluations are initially performed every month, then every three months and, finally, every six months [8,18], the reproducibility of the titrations must be maintained for up to six months. Our results demonstrated concordant titrations in all the cases for up to three years and in almost all of them for six years, allowing the safe use of these procedures. 

The other indication of the use of stored serum samples in biobanks is the study of new aspects of PCM, such as criteria of cure or biomarkers for recidivation or relapse, the latter one observed in 5% of the cases, after appropriate treatment [21]. These studies usually demand a number of patients higher than that admitted every year, which is 15 in our service [22]. Thus, an organized bank of clinical data, from admission to discharge, associated to serum samples stored at -80^o^C should permit the evaluation of new parameters in a shorter period of time and consequently with a lower cost. As the progress of the serological evaluation is one of the main criteria of cure of PCM, its results are frequently used in studies of any variable along the treatment [8,18,23]. Therefore, it is of great importance the evaluation of the effect of storage at -80^o^C on the serum levels of specific anti-*P. brasiliensis* antibodies, which is the aim of the present study. 

The studies focusing the effect of the storage of serum samples on the reproducibility of the titration of antibodies or antigens are scarce.

The detection of galactomannan in the serum of patients with risk to develop invasive aspergillosis (IA) was evaluated using the enzyme immune assay *Aspergillus* *imunoenzimático galactomanano*(*GM EIA*) [24,25]. This study evaluated serum and plasma-EDTA samples stored at 4^o^C until titration, in about 72 hours, and after storage for about one year at the same temperature. In addition, the effect of the freeze-thaw cycle on the reproducibility of the titers was evaluated in plasma samples stored at -80^o^C for one week. The retest demonstrated a decrease in the positivity in 17% of the serum samples, predominantly from patients with probable or possible IA, whose previous results were considered false-positive. Only two samples tested negative at test and positive at retest. The inverse correlation between albumin concentration and enzyme immune assay could explain these results. The study of plasma samples showed no influence of the storage at 4^o^C, nor after frozen at -80ºC [25]. The authors draw attention to the stability of the plasma samples, probably due to the inhibition of blood enzymes, caused by EDTA. 

In a recent study, the titers of antigens from *Cryptococcus* species determined by the semi-quantitative method lateral flow assay - LFA in stored serum were compared with those determined at the moment the blood was drawn, by the traditional qualitative LFA method using multiple repetitions in serum samples with progressive dilutions [26]. In the discussion of the results, the authors register the use of stored serum as a limitation of the study because the freeze-thaw cycle could have caused some discrepancy in the titer or in the reading of traces of positivity. There was not any study that had evaluated the influence of the storage on the results of cryptococcal antigens determination.

Both studies presented above were related to the titration of antigens and not to antibodies, which is the aim of the present evaluation. In our study, the antibody serum levels were higher in patients with AF than those with CF, confirming previous findings [6]. The comparison of the distribution of titers between test and retest showed that the storage can lead to some alterations of titers. However, the persistence of the titration for up to six years in serum samples stored at -80°C allows its use in evaluations to be carried out in this period.

This study also shows that in serum from patients with AF as well as those with CF, the difference between test and retest were of one dilution, which in the clinical practice is considered a concordant titration. This level of difference can be observed in evaluations of the same serum sample, performed by skilled researchers from two laboratories, using the same antigen [27]. However, differences higher than one dilution were observed in some samples, being clinically relevant. In addition, the comparison of the titers determined in the test with those in the retest showed no predominance of any one, which renders impossible the association of these findings with the freeze-thaw cycle. 

The conversion of serum samples from reagent in the test to non-reagent in the retest, which was observed in storage for more than six years, is an outstanding finding because it changes completely the clinical interpretation. These data reinforce the suggestion to use serum samples stored for no more than six years. 

Studies with proteomic methodology, such as those carried out by Sylvestre et al. [20], could elucidate the influence of the storage by comparing test and retest findings. However, this research design requires further investigation. In addition, it is of great importance to select the most appropriate conditions for the storage of clinical specimens, aiming to preserve its quality and to permit its use in future studies. For this reason, the antibody levels evaluated by the double agar gel immunodiffusion test were determined in different blood matrices. 

Our prospective study showed that the levels of anti-*P. brasiliensis* antibodies were not different as to the blood matrix used - serum, plasma obtained with EDTA or with heparin, in any of the stages analysed, i.e., at the moment the blood was drawn and after two, four and six months of storage at -20^o^C. These titrations also showed direct correlation when the blood matrices were evaluated 2X2, in all the stages studied. The reproducibility of the antibody titers observed in this experiment, carried out with the same antigen and by the same technician, permitted an accurate evaluation of the storage effect. 

The biochemical composition of plasma and serum present differences: the plasma contains albumin, immunoglobulin G (IgG), acid α_1_-glycoprotein, IgA, IgM, transferrin, haptoglobin, α_1_-antitripsin, α_2_-macroglobulin, apolipoprotein A-I, apolipoprotein A-II and fibrinogen while in the serum the albumin constitutes 55% of the protein content, in addition to IgG, IgA, haptoglobin, transferrin and α_1_-antitripsin, which constitute about 85% of the total proteome [20]. In spite of these differences, serum and plasma are preserved in a similar way for at least six months [28,29,30]. This was the time of storage evaluated in the present study, for different blood matrices. Future studies should evaluate the influence of longer periods of storage.

Another aspect that needs to be investigated is the possible influence of different antifungal compounds in the blood matrices. Amphotericin B was identified in blood samples from patients with systemic mycoses, stored at -10^o^C for up to nine months. The possible action of this antifungal compound could be speculated, introducing another parameter in the evaluation of the circulating antibody stability [31]. 

The choice of the blood matrix is related to the type of molecule that should be identified. Wang et al. [32] evaluated serum and plasma for the identification of microRNAs as biomarkers and observed a higher amount of miRNA in the first one. However, at evaluating the formation of serum and plasma, they observed that the coagulation process provided a stressful environment that would lead to the stimulation of the miRNA identification, altering the quantification of the biomarker of interest. Thus, the plasma was considered the best clinical material to study miRNA.

Our findings suggest that the serum should be the blood matrix of choice, because of its long availability and the maintenance of the present matrix. In addition, it was demonstrated that plasma samples are good matrices for storage at -20^o^C. 

## Conclusion

In conclusion, serum samples stored at -80^o^C up to six years show few discordant results, and plasma samples are good matrices for storage at -20^o^C. The 6-month period of storage at -20^o^C for plasma samples is the limitation of the study. As the study time should be longer than six years, it will be performed in another project.

## Supplementary material

The following online material is available for this article:

Additional file 1. Titers of specific serum antibodies determined by double agar gel immunodiffusion test, in samples from 20 patients with the acute/subacute form and 160 with the chronic form of paracoccidioidomycosis. Distribution of the titers determined at the moment of blood draw (test) and after at least six months of storage at -80^o^C (retest), regarding clinical form. Frequencies were compared by the Goodman test.

## References

[B1] Teixeira MM, Theodoro RC, Carvalho MJA, Fernandes L, Paes HC, Hahn RC, Mendoza L, Bagagli E, San-Blas G, Felipe MSS (2009). Phylogenetic analysis reveals a high level of speciation in the Paracoccidioides genus. Mol Phylogenet Evol.

[B2] Restrepo A (1985). The ecology of Paracoccidioides brasiliensis: a puzzle still unsolved. Sabouraudia.

[B3] Marques SA, Franco MF, Mendes RP, Silva NC, Baccili C, Curcelli ED, Feracin AC, Oliveira CS, Tagliarini JV, Dillon NL (1983). Epidemiological aspects of paracoccidioidomycosis in the endemic area of Botucatu (São Paulo - Brazil). Rev Inst Med Trop Sao Paulo.

[B4] Franco M, Bagagli E, Scapolio S, Silva Lacaz C (2000). A critical analysis of isolation of Paracoccidioides brasiliensis from soil. Med Mycol.

[B5] De Albornoz MB (1971). Isolation of Paracoccidioides brasiliensis from rural soil in Venezuela. Sabouraudia.

[B6] Moreto TC, Marques MEA, Oliveira MLSC, Moris DV, Carvalho LR, Mendes RP (2011). Accuracy of routine diagnostic tests used in paracoccidioidomycosis patients at a university hospital. Trans R Soc Trop Med Hyg.

[B7] Lauand F (1966). Contribuição para o estudo da morfologia do Paracoccidioides brasiliensis nos tecidos orais. Rev Inst Med Trop São Paulo.

[B8] Mendes RP, Cavalcante RS, Marques SA, Marques MEA, Venturini J, Sylvestre TF, Paniago AMM, Pereira AC, Silva JF, Fabro AT, Bosco SMG, Bagagli E, Hahn RC, Levorato AD (2017). Paracoccidioidomycosis: current perspectives from Brazil. Open Microbiol J.

[B9] Biagioni L, Souza MJ, Chamma LG, Mendes RP, Marques SA, Mota NG, Franco M (1984). Serology of paracoccidioidomycosis. II. Correlation between class-specific antibodies and clinical forms of the disease. Trans R Soc Trop Med Hyg.

[B10] Benard G, Romano CC, Cacere CR, Juvenale M, Mendes-Giannini MJ, Duarte AJ (2001). Imbalance of IL-2, IFN-gamma and IL-10 secretion in the immunosuppression associated with human paracoccidioidomycosis. Cytokine.

[B11] Dee TH, Berger CS (1979). Stability of Anti-Candida Precipitating Antibody in Stored Sera as Determined by Counterimmunoelectrophoresis. J Clin Microbiol.

[B12] Taschdjian CL, Kozinn PJ, Cuesta MB, Toni EF (1972). Serodiagnosis of candidal infections. AJCP.

[B13] Lau KS, Yeung C, Carlsten C (2020). Stability of serum precipitins to Aspergillus fumigatus for the diagnosis of allergic bronchopulmonary aspergillosis. Allergy Asthma Clin Immunol.

[B14] Woodcock JP (1976). Physical properties of blood and their influence on blood-flow measurement. Rep Prog Phys.

[B15] Sociedade Brasileira de Patologia (2010). Recomendações da Sociedade Brasileira de Patologia Clínica: Medicina Laboratorial para coleta de sangue venoso.

[B16] Yu Z, Kastenmüller G, He Y, Belcredi P, Möller G, Prehn C, Mendes J, Wahl S, Roemisch-Margl W, Ceglarek U, Polonikov A, Dahmen N, Prokisch H, Xie L, Li Y, Wichmann H, Peters A, Kronenberg F, Suhre K, Adamski J, Illig T, Wang-Sattler R (2011). Differences between human plasma and serum metabolite profiles. PLoS One.

[B17] Mannello F (2008). Serum or plasma samples? The "Cinderella" role of blood collection procedures: preanalytical methodological issues influence the release and activity of circulating matrix metalloproteinases and their tissue inhibitors, hampering diagnostic trueness and leading to misinterpretation. Arterioscler Thromb Vasc Biol.

[B18] Mendes RP, Franco M, Lacaz CS, Restrepo-Moreno A, Del Negro G (1994). Paracoccidioidomycosis.

[B19] Zar JH (2010). Biostatistical analysis.

[B20] Restrepo MA (1966). La prueba de immunodiffusion en el diagnostico de la paracoccidioidomicosis. Sabouraudia.

[B21] Sylvestre TF, Cavalcante RS, Silva JF, Paniago AMM, Weber SS, Pauletti BA, de Carvalho LR, Santos LD, Mendes RP (2018). Serological proteomic biomarkers to identify Paracoccidioides species and risk of relapse. PLoS One.

[B22] Azevedo PZ, Cavalcante RS, Mendes RP (2015). Paracoccidioidomycosis. Incidence of new cases in the period 1988 - 2014. Distribution as to clinical form.

[B23] Benard G Hong MA, Del Negro GM Batista L, Shikanai-Yasuda MA Duarte AJ (1996). Antigen-specific immunosuppression in paracoccidioidomycosis. Am J Trop Med Hyg.

[B24] Guitard J, Sendid B, Thorez S, Gits M, Hennequin C (2012). Evaluation of a recombinant antigen-based enzyme immunoassay for the diagnosis of noninvasive aspergillosis. J Clin Microbiol.

[B25] Kimpton G, White PL, Barnes RA (2014). The effect of sample storage on the performance and reproducibility of the galactomannan EIA test. Med Mycol.

[B26] Skipper C, Tadeo K, Martyn E, Nalintya E, Rajasingham R, Meya DB, Kafufu B, Rhein J, Boulware DR (2020). Evaluation of serum Cryptococcal antigen testing using two novel semiquantitative lateral flow assays in persons with Cryptococcal antigenemia. J Clin Microbiol.

[B27] Collachiti Moreto T, Pardini Vicentini Moreira A, Noronha-Passos A, Sayuri Kohara V, Carvalho LR, Poncio Mendes R (2009). Serological diagnosis of paracoccidioidomycosis. Evaluation of negative serum samples on immunodiffusion test from patients with confirmed disease. Rev Soc Bras Med Trop.

[B28] Lee PY, Osman J, Low TY, Jamal R (2019). Plasma/serum proteomics: depletion strategies for reducing high-abundance proteins for biomarker discovery. Bioanalysis.

[B29] Keshishian H, Burgess MW, Specht H, Wallace L, Clauser KR, Gillette MA, Carr AS (2017). Quantitative, multiplexed workflow for deep analysis of human blood plasma and biomarker discovery by mass spectrometry. Nat Protoc.

[B30] Qian W, Kaleta DT, Petritis BO, Jiang H, Liu T, Zhang X, Mottaz HM, Varnum SM, Camp DG, Huang L, Fang X, Zhang W, Smith RD (2008). Enhanced detection of low abundance human plasma proteins using a tandem IgY12-SuperMix immunoaffinity separation strategy. Mol Cell Proteomics.

[B31] Fields BT, Bates JH, Abernathy RS (1970). Amphotericin B serum concentrations during therapy. Appl Microbiol.

[B32] Wang K, Yuan Y, Cho J, McClarty S, Baxter D, Galas DJ (2012). Comparing the MicroRNA spectrum between serum and plasma. PLoS One.

