# Supplementary material for: Reproducibility of double agar gel immunodiffusion test using stored serum and plasma from paracoccidioidomycosis patients
Source: J Venom Anim Toxins Incl Trop Dis. 2023 Jan 16;29:e20220045. doi: 10.1590/1678-9199-JVATITD-2022-0045 (PMC9842191; doi:10.1590/1678-9199-JVATITD-2022-0045)
Supplement: Additional file 1. [file 1678-9199-jvatitd-29-e20220045-s1.pdf]

J Venom Anim Toxins incl Trop Dis

https://doi.org/10.1590/1678-9199-JVATITD-2022-0045

Supplementary Material to “Reproducibility of double agar gel immunodiffusion test using stored serum and plasma from paracoccidioidomycosis patients”

Additional file 1 Titers of specific serum antibodies determined by double agar gel immunodiffusion test, in samples from 20 patients with the acute/subacute form and 160 with the chronic form of aracoccidioidomycosis. Distribution of the titers determined at the moment of blood draw (test) and after at least six months of storage at –80oC (retest), regarding clinical form. Frequencies were compared by the Goodman test

**Results**	**Acute form**		**Chronic form**	
	**TEST**	**RETEST**	**TEST**	**RETEST**
**Titers***	**Number (%)**		**Number (%)**	
Non-reagent	– (0.0)	4 (20.0)	05 (3.1)A	30 (18.8)A
**Reagent**				
Undiluted	– (0.0)	– (0.0)	17 (10.6)A	9 (5.6)AB
2	1 (5.0)	– (0.0)	14 (08.8)A	2 (1.3)B
4	2 (10.0)	4 (20.0)	24 (15.0)A	2 (1.3)B
8	– (0.0)	– (0.0)	16 (10.0)A	13 (8.10)AB
16	2 (10.0)	1 (5.0)	22 (13.8)A	19 (11.9)AB
32	1 (05.0)	– (0.0)	20 (12.5)A	18 (11.3)AB
64	2 (10.0)	1 (5.0)	22 (13.8)A	8 (5.0)AB
128	3 (15.0)	3 (15.0)	14 (8.8)A	23 (14.4)AB
256	4 (20.0)	– (0.0)	04 (2.5)A	11 (6.9)AB
512	5 (25.0)	3 (15.0)	– (0.0)B	17 (10.6)AB
1024	– (0.0)	4 (20.0)	02 (1.3)A	8 (5.0)AB
Total	20 (100.0)		160 (100.0)	

Capital letters compare frequencies in the same column. Frequencies not followed by letters or followed by the same letter do not differ (**p** > 0.05), while those followed by different letters show a statistically significant difference (**p** ≤ 0.05). *Titers are the inverse of the dilution.
